# A mini-review on the effects of COVID-19 on younger individuals

**DOI:** 10.1177/1535370220975118

**Published:** 2020-11-19

**Authors:** Madhumitha Manivannan, Manasi P Jogalekar, Muthu Subash Kavitha, Balu Alagar Venmathi Maran, Prakash Gangadaran

**Affiliations:** 1 Elgin Academy, Elgin, IL 60120, USA; 2 Brigham and Women’s Hospital, Harvard Medical School, Boston, MA 02115, USA; 3 Graduate School of Advanced Science and Engineering, Hiroshima University, Higashi Hiroshima, Hiroshima 739-8511, Japan; 4 Borneo Marine Research Institute, Universiti Malaysia Sabah, Jalan UMS 88400, Kota Kinabalu, Sabah, Malaysia; 5 Department of Nuclear Medicine, School of Medicine, Kyungpook National University, Daegu 41944, Republic of Korea; 6 BK21 Plus KNU Biomedical Convergence Program, Department of Biomedical Science, School of Medicine, Kyungpook National University, Daegu 41944, Republic of Korea

**Keywords:** COVID-19, younger individuals, SARS-CoV-2, immune system, psychology, education

## Abstract

Coronavirus disease 2019 (COVID-19) pandemic has uprooted our lives like never before since its onset in the late December 2019. The world has seen mounting infections and deaths over the past few months despite the unprecedented measures countries are implementing, such as lockdowns, social distancing, mask-wearing, and banning gatherings in large groups. Interestingly, young individuals seem less likely to be impacted by severe acute respiratory syndrome coronavirus 2 (SARS-CoV-2), the virus responsible for COVID-19. While the rate of transmission, symptom presentation, and fatality is lower in children than people from other age groups, they have been disproportionately affected by strict lockdown measures needed to curb viral spread. In this review, we describe the association between patient age and COVID-19, epidemiology of SARS-CoV-2 infection in children, psychological effects associated with lockdowns and school closures, and possible mechanisms underlying lower transmission rate of COVID-19 in children.

## Impact statement

The impact of COVID-19 on children is a relatively unexplored area, with only limited information available in terms of disease transmission, fatality, and symptomatic disease burden in children. During the initial few months of the pandemic, very few confirmed cases were reported in children, most of which were due to a close contact with an infected family member. That picture seems to have changed during the recent months with as much as 20% of confirmed cases reported in young adults. This underscores the need to follow social distancing guidelines, which may play a role in preventing community transmission. On the other hand, it is important to consider the impact of school closures and lockdowns on the lives of young children. We hope that this article will shade some light on potential measures we could take to ease the psychological burden associated with social distancing, in young individuals.

## Introduction

Coronavirus disease 2019 (COVID-19) pandemic has adversely affected older population around the world, especially those over the age of 55.^1^ While there is extensive literature relating to the effects of COVID-19 on these demographics, little to no consolidated information is available regarding the impact of COVID-19 on children and adolescents. Even if both parents have COVID-19, a child can remain relatively unscathed by maintaining reasonable distance (i.e. in the same house) from them. Previous pandemics such as the 1918 Spanish Influenza outbreak, which posed many problems (excluding war) to the American Healthcare landscape, were found to hit the younger populations the most. The fact that COVID-19 does not do so poses the question as to what younger generations may have that older generations do not, to help them fight COVID-19. COVID-19 is caused by a novel beta-coronavirus family member called severe acute respiratory syndrome coronavirus 2 (SARS-CoV-2).^2,3^ In December 2019 (and potentially earlier, though unrecognized), SARS-CoV-2 emerged as a peculiar pneumonia-causing virus in Hubei province, China, most likely as a result of natural selection in animal hosts (bats, pangolins) prior to zoonotic transfer. There are now seven members of this viral family that are known to infect humans with three having the potential to cause a severe respiratory disease. The two outbreaks preceding SARS-CoV-2 include the first SARS virus emerging in the late 2002 in Guangdong province, China (now referred to as SARS-CoV-1), and the Middle-East Respiratory Syndrome coronavirus (MERS-CoV) in 2012 in Saudi Arabia. SARS-CoV-2 has rampaged through various cities in the world since the beginning of 2020; the World Health Organization (WHO) declared it a Public Health Emergency of International Concern on 30 January 2020, and a pandemic on 7 March 2020. At the time of writing this review (November 2020), ∼54 million individuals in at least 215 countries were infected, and >1.3 million had died due to the virus.

## Age and COVID-19

Accumulating evidence has unravelled several COVID-19-related health disparities including age, race, and overall health of the individual. Older individuals, particularly those >50, are at high risk of contracting COVID-19, and have poor prognosis compared to those from other age groups, possibly due to pathophysiological changes associated with aging.^2^ As a result, mortality rate tends to be higher in this population.^4,5^

A recent study involving both younger and older COVID-19 patients (n = 221) demonstrated longer treatment durations and frequent respiratory failures in patients ≥60 years of age, indicating higher degree of severity in this population. In addition, older individuals were also less likely to respond to treatments the same way younger people do, due to their weak immune system.^6^ Interventions such as corticosteroids, antibiotics therapy, and ventilation were routinely used to treat older patients.^6^ Even then, the cure rate remained relatively lower than that in younger population.^6^

Of late, there is an indication that COVID-19 age distribution may have changed during the summer months. Recent reports suggest higher number of COVID-19 infections (>20% of all positive cases) among younger adults (aged 20–39 years) during June–August period than that observed at the beginning of the pandemic, indicating their possible role in community transmission. This is another reminder for all of us to follow appropriate social distancing protocols, wear masks, and avoid in-person meetings with large groups. These actions will not only result in the well-being of younger population but will also be instrumental in reducing transmission to high-risk populations.^7^

## Effects of SARS-CoV-2 infection in younger individuals

### Epidemiology of SARS-CoV-2

Limited information in the literature suggests that younger individuals are less likely to be affected by COVID-19, albeit for unknown reasons.^8^ There is a scarcity of data relating to the prevalence of COVID-19 in children due to the lack of extensive testing at the beginning of the pandemic.^9^ According to a retrospective study, the rate of positive tests was 1.6% among young individuals (<16 years of age) suffering from respiratory tract infections in early January in Wuhan.^10^ By the end of January and early February, that rate was down to 0.6%^11^ and 0.8%,^12^ respectively, most cases being associated with the household contact with an infected family member. By mid-February, the proportion of confirmed cases in adolescents (10–19 years of age) was found to be slightly higher (1.2%; 1 death) than that observed in younger children (0.9%).^13^ At the same time, the rate of positive cases in children remained high in China (3.5%).^9^ A few pediatric cases were also reported by countries such as Singapore, Malaysia, Korea, Germany, Italy, Australia, and Vietnam.^14^ By the end of February, 2.4% of total confirmed cases were reported in young individuals (<19 years of age), although most of the children showed mild symptoms.^8^ As of mid-March, the rate of positive cases was much higher (5.2%) in adolescents (10–19 years of age) compared to that (1%) in children (0–9 years), although no deaths were reported.^14^Summer months from March through September have unraveled more disturbing pattern, when more than 270,000 positive cases were confirmed in children, out of which 63% were adolescents and 37% were school-aged children.Leeb RT, Price S, Sliwa S, Kimball A, Szucs L, Caruso E, et al. COVID-19 trends among school-aged children—United States, March 1–September 19, 2020. Morbidity and Mortality Weekly Report 2020;69(39):1410.^15^ A 36-h-old newborn was the youngest child reported to have contracted COVID-19.^16^

One of the most prominent symptoms observed in COVID-19-positive young individuals is multisystem inflammatory syndrome in children (MIS-C). While the pathophysiology of MIS-C still remains unclear, the condition may be the result of an excessive immune response characterized by the generation of non-specific antibodies against an array of respiratory viruses (excluding SARS-CoV-2).^17^ Symptoms associated with MIS-C include fever, rash, gastrointestinal issues, and myocarditis, a cardiac muscle inflammation characterized by high circulatory concentrations of ferritin, troponin, and D-dimer.^17^

### Recovery and mortality

Most of the young individuals were either asymptomatic or showed mild symptoms, resulting in a fewer hospitalizations,^18^ for reasons currently unknown. A few studies and a hypothesis may particularly answer this question. Several studies suggest that young individuals are susceptible to contracting various viral airway infections, and <75% of all young individuals already contracted one of the seasonal coronavirus infections before the age of 4. The higher titre of anti-seasonal coronavirus (CoV) antibodies in a young individual may eliminate SARS-CoV-2 faster than elderly.^19–21^ Another potential explanation could be the expression of transmembrane angiotensin-converting enzyme 2 (ACE2), a key cellular receptor that facilitates SARS-CoV2 entry into cells, thereby causing infection.^22^ A previous study suggests that ACE2 expression is highest in younger people when compared to that found in older individuals, and lower ACE2 expression is associated with poor clinical outcome.^23^ A few recent studies indicate that live attenuated vaccines such as measles or Bacillus Calmette-Guérin (BCG) may protect against COVID-19.^24^ A young individual may be protected against SARS-CoV-2 due to age-related factors such as heterologous immune responses and a higher number of memory T cell when compared to the elder persons.^25^ The mortality rate of in younger individuals (<19 years) in Republic of Korea, China, and Italy were 0%, 0.2%, and 0% respectively.^26^ These studies suggest that younger individual can easily clear the virus from the system, showing mild or no symptoms.

### Psychological effect

The COVID-19 pandemic has also brought forth a new wave of panic and confusion from the general public. This negative effect can be seen in the younger generations. Many studies in this topic have suggested that the psychological burden caused by COVID-19 was seen in other pandemic situations as well, such as the response of young Chinese individuals during the SARS outbreak, or the Influenza A (H1N1) outbreak.^27^ The most prominent reason for worry and panic lies in the fear of getting infected, which can be good in terms of making the general population follow the social distancing rules, but worse for the individuals themselves.^28^ The long-term effects of continuously staying at home have heightened the effects of hysteria and paranoia, due to the lack of stimulation inside a home. A sharp uptick in the number of accidental injuries and suicides has been reported in younger individuals during school closures, indicating their profound impact on mental health of children.^17^ This type of suppressive environment can lead to a desire to do potentially harmful things outside of the recommended sphere of safety.^29^ Taking away what people have once enjoyed (i.e. meeting up with friends in real life, shopping, going on trips, etc.), and the tightened restrictions enforced by the families of minors (i.e. not going out except for some approved activities, or interrogating them on their whereabouts), have led many individuals to feel powerless. As a result, they often feel less hopeful for the future as this pandemic strikes harder, affecting countries such as the USA, India, and several European countries.^30^ The effects of the stay-at-home order have been accelerated by the advent of social media, where seeing role models in a more personal setting can greatly impact a person’s perception of the issue. In many instances, popular accounts on social media serve as a double-edged sword—they can be used to encourage important issues, or instigate harmful behavior. For example, a recent event involving a handful of “influencers” showed the lack of mask-wearing and social distancing. These influencers were heard saying that they “do not care” in the slightest, thereby making themselves look more appealing by taking risks. Since they have a large following of mostly young, aspirational individuals, this kind of content can harm decision-making process on many levels, especially when trying to host large gatherings or even meeting with other people outside.^31^ This effect should be emphasized by celebrities in the social media realm, as they know exactly what kind of audience they have. This will create awareness in the general public regarding the appropriate social distancing protocols they should follow.

### Education

One way in which school closures may prove successful during outbreaks is by requiring parents to work from home, thereby reducing interactions related to the job. Previous studies have noted, however, the negative consequences of closing schools, including economic losses to working parents, health care staff and other main employees being diverted from work to childcare and to society due to lack of parental profitability, lack of education, child health risks, especially among the most needy students, and dietary issues specifically for children for whom free school meals are a significant nutritional source.^32^ In itself, social isolation carries a number of psychological damages. A brief analysis showed that children’s behavior changed, and their social interactions declined amid unforeseen school closures but did not stop, with some indication that this was notably so for older children and those whose parents objected with closures.^33^ By March 2020, many countries had instituted large-scale or national school closures to reduce COVID-19 transmission. There are potential explanations as to why school suspensions during COVID-19 pandemic could be less successful than those enforced during influenza outbreaks. Children contribute more to influenza spread than adults, due to their weaker immune system and elevated infection rates related to symptomatic illness.^32^ On the other hand, during the COVID-19 pandemic, the rate of transmission in children tends to be much lower than that predicted from their demographic, although evidence for this is inconsistent. Some reports indicate that children may be as likely to be affected as adults but remain relatively asymptomatic or have a mild form of the infection.^34^ The question remains whether the small proportion of confirmed COVID-19 cases in children in mainland China is due to a decreased risk of infection, subclinical or milder infection, or particular demographic variables (e.g. one-child policy). There is currently no evidence of COVID-19 transmission through child-to-child interaction or via schools, although family transmission has an important role in the outbreak.^33^

### Future perspectives

The treatment options for COVID-19 are still limited, and there are no specific vaccines available till date. Several clinical trials are currently undergoing, where the interventions such as antiviral medications,^35,36^ immunoglobulins,^37^ high-flow nasal oxygen,^38^ and ventilation^39,40^ are being used alone or in combination, to determine their efficacy against COVID-19. The best way to stop the spread of the virus is through self-isolation and avoiding contact with COVID-19 patients. Due to the lockdown and closure of educational institutions, the younger individuals are less likely to get exposed to SARS-CoV-2. Nevertheless, younger individuals may still be easily infected through family members, who get infected due to their exposure to external environment.

## Conclusions

Although COVID-19 predominantly targets elderly populations due to the lack of defences by a deteriorating immune system, underlying diseases, and lack of vaccinations, the lower rate of transmission and symptomatic disease burden in children and adolescents is puzzling and warrants further investigation. This could be attributed to elevated expression of ACE2 in upper respiratory tract or the absence of excessive immune response (e.g. cytokine storm) commonly observed in elderly patients. In order to prevent the spread of disease, strict social distancing protocols have been mandated, posing challenges in childcare. The psychological burden that comes with school closures and access to the social media also impacts mental health in young individuals. Evidence remains inconclusive as to whether such drastic measures as school closures are helpful in reducing the risk of transmission.
